# Effect of manual OCTA segmentation correction to improve image quality and visibility of choroidal neovascularization in AMD

**DOI:** 10.1038/s41598-024-61551-z

**Published:** 2024-06-18

**Authors:** Daniel N. Deussen, Anna Heinke, Wyatt Elsner, Carlo Miguel B. Galang, Fritz Gerald P. Kalaw, Alexandra Warter, Dirk-Uwe Bartsch, Lingyun Cheng, William R. Freeman

**Affiliations:** 1 Jacobs Retina Center, 9415 Campus Point Drive, La Jolla, CA 92037 USA; 2 Viterbi Family Department of Ophthalmology and Shiley Eye Institute, University of California San Diego, 9415 Campus Point Drive, La Jolla, CA 92037 USA; 3 Department of Ophthalmology, University Hospital, Ludwig-Maximilians-University, 80336 Munich, Germany; 4 The Department of Cognitive Science, University of California San Diego, San Diego, USA

**Keywords:** Medical imaging, Diagnostic markers

## Abstract

In this retrospective case series on neovascular age-related macular degeneration (nAMD), we aimed to improve Choroidal Neovascularization (CNV) visualization in Optical Coherence Tomography Angiography (OCTA) scans by addressing segmentation errors. Out of 198 eyes, 73 OCTA scans required manual segmentation correction. We compared uncorrected scans to those with minimal (2 corrections), moderate (10 corrections), and detailed (50 corrections) efforts targeting falsely segmented Bruch’s Membrane (BM). Results showed that 55% of corrected OCTAs exhibited improved quality after manual correction. Notably, minimal correction (2 scans) already led to significant improvements, with additional corrections (10 or 50) not further enhancing expert grading. Reduced background noise and improved CNV identification were observed, with the most substantial improvement after two corrections compared to baseline uncorrected images. In conclusion, our approach of correcting segmentation errors effectively enhances image quality in OCTA scans of nAMD. This study demonstrates the efficacy of the method, with 55% of resegmented OCTA images exhibiting enhanced quality, leading to a notable increase in the proportion of high-quality images from 63 to 83%.

## Introduction

Age related macular degeneration (AMD) is a major cause of severe visual impairment in individuals above the age of 55 years^1^. The traditional classification of AMD encompasses both neovascular AMD (nAMD), also known as wet AMD, and the dry type (dAMD). Despite sharing similarities in genetics and pathophysiological changes, the treatment approaches for these two types differ substantially^2^. Thus, distinguishing between the two types is crucial, as prompt and appropriate treatment offers the best prospects for maintaining vision in nAMD patients^3^. The primary distinction lies in the formation of choroidal neovascularization (CNV), a severe complication and defining feature of nAMD, which can result in acute and often irreversible vision loss due to active leakage. The leakage of CNV is typically diagnosed through fundoscopy and fluorescein angiography, which have served as the gold standard in clinical practice for decades^4^.

Optical coherence tomography angiography (OCTA) is a non-invasive imaging modality that can provide high-resolution images of the retinal vasculature and is increasingly being used to diagnose and monitor AMD^5^. OCTA is gaining popularity in ophthalmological clinical practice as well as in clinical research, where it helps to better understand the pathophysiology of different vascular diseases. It offers relatively quick and easy acquisition, good reproducibility, and no need for an intravenous dye that bears possible side effects^6^. Especially for CNV visualization OCTA is very helpful, as the choroidal microvasculature can be well displayed and reviewed in one single *en face* image. As quality and clinical experience with OCTA are increasing, sensitivity and specificity for CNV detection with combined OCTA *en face* and cross-sectional analysis are almost reaching those of fluorescein angiography as the accepted gold standard^4^.

In general, image quality and automatic layer segmentation are only moderately accurate in modern OCTA devices with errors in segmentation being most common where there is a distortion of anatomy such as in CNV. Different factors can decrease image quality, i.e., opacities of the optical media, patient compliance, and especially larger retinal lesions. Thus, misaligned retinal layer segmentation often appears in areas of pathology, possibly leading to misdiagnosis. A need for manual correction of these segmentation errors has been described and a need for manual correction has been shown in both nAMD^7,8^ and other retinal diseases such as diabetic macular edema (DME)^9^. When different pathologies were compared, nAMD showed the highest rate of false segmentation compared to other retinal pathologies^10^. Nevertheless, resegmentation and segmentation errors are discussed in only a minority of studies on the clinical relevance of OCTA to retinal disease (Falavarjani et al.) with around 12% of published studies stating that they manually corrected their OCTAs images^11^.

Segmenting all retinal layers in a large volume of scans, such as the 512 scans from the Heidelberg Spectralis, is extremely laborious and time-consuming, thus there is a need for more robust and faster methods. For this reason, we wished to determine the minimum number of manual segmentation corrections required to enhance the visualization of choroidal neovascularization (CNV) in OCTA. This is especially pertinent when dealing with inaccurate automated segmentation using Heidelberg Spectralis (SD-OCT) while still ensuring practicality in the daily clinical routine. Stromer et al. suggested a similar approach of “user-assisted” correction but tested it on a prototype SS-OCT and did not focus on CNV detection in particular^12^.

Relevant and reproducible correction approaches are still lacking in the literature. This study aims to provide a quick and simple guide for manual segmentation correction to improve OCTA image quality and reliability.

## Methods

### Examinations and procedures

This study was approved by the Institutional Review Board of the University of California San Diego in California (#120516), USA, and complied with the Health Insurance Portability and Accountability Act of 1996. Informed consent was obtained from all the subjects. All the data that were collected were anonymized for patient’s safety. Data collection and analysis were conducted according to the Principles of the Declaration of Helsinki.

A retrospective electronic health record (EHR) analysis to search for patients diagnosed with neovascular AMD was conducted using EPIC. Two retina specialists then based on diagnosis found in EHR reviewed 198 consecutive OCTA scans using Heyex 2 platform for quality of the scan, visibility of CNV vessels, background quality, image noise and artifacts.

OCTA was performed using the Heidelberg HRA + OCT Spectralis System version 1·11·2·0 (Heidelberg Engineering, Heidelberg, Germany). OCTA was centered in the fovea or for non-central CNV on the lesion. A 10 × 10 degrees field of view, consisting of 512 consecutive B-scans were obtained. Inclusion criteria included scans of patients with diagnosis of neovascular AMD (newly diagnosed, undergoing treatment or in remission), scans that required manual segmentation correction of Bruch’s Membrane (BM) segmentation errors in OCT adjacent to OCTA that is in cases where automated segmentation by Heidelberg software falsely delineated this retinal layer in OCT, as well as scans with background noise. Exclusion criteria included: scans with good automated layer segmentation of Bruch’s membrane, poor quality of OCTA scans defined as scans with quality index Q < 25, other causes of CNV (such as idiopathic, myopic, traumatic, etc.), and comorbid conditions that obscure the media like cataracts and cornea opacities.

Each patient during their visit in the retina clinic, underwent a comprehensive ophthalmologic examination, anterior segment examination using slit lamp biomicroscope (Haag-Streit, USA), and dilated fundus examination using an indirect ophthalmoscope. Further intraocular pressure was measured using the iCare TA01i Tonometer (Icare, USA, Inc.), and the best-corrected visual acuity (BCVA) was obtained, using the Early Treatment Diabetic Retinopathy Study (ETDRS) chart.

### Manual OCTA correction

**Figure 1:**
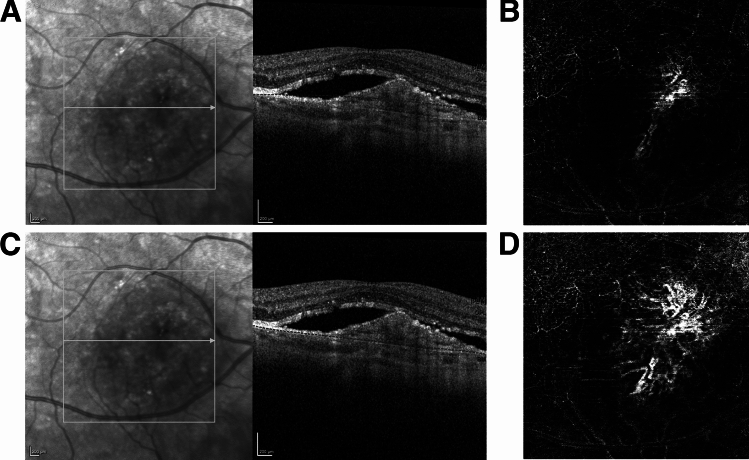
Manual segmentation layer correction of Bruch’s Membrane (BM). Review of the corresponding OCTA B-scan (Image **A**) shows the major error in the segmentation of BM, where the machine software misidentified it and displaced the BM line superiorly. Image C shows the same location of the BM line after the segmentation error was manually corrected (Image **C**). The manually corrected data is subsequently saved, followed by the execution of the software propagation. Image (**B**) shows choroidal neovascularization (CNV) with BM segmentation error, and image (**D**) showed improved visualization of CNV after manual correction of segmentation error.

Out of 198 assessed eyes with OCTA scans, 78 showed major segmentation errors of BM. Two retina specialists performed manual segmentation correction of Bruch’s Membrane (BM) layer in an OCT-B scan adjacent to OCTA. In the Heidelberg software, edit layer segmentation was selected, the BM was chosen, and segmentation errors were manually corrected. The BM is situated between the RPE and the choriocapillaris. Either the drag and drop nodes or the circle tool was used to modify the BM segmentation. Each 512- line uncorrected OCTA scan was exported as an .e2e file from Heyex-2. An uncorrected scan was used as a baseline to which to compare resegmented images. Two, ten, and fifty of the 512 B scan images were corrected for BM location in each exported OCTA sample. Correction was carried out evenly distributed within the area of segmentation error. Areas of correct segmentation were left unchanged. The manual correction was automatically propagated by a propagation tool embedded in Heidelberg Software to resegment all the 512 B scans (Fig. 1).

### Expert grading of corrected OCTA

OCTA *en face* slabs were presented to two retina experts, additionally, the OCT B scans of the region of interest were provided for every OCTA graded. The image generated after OCTA segmentation correction was graded compared to the uncorrected image as a baseline.

A grading as follows was utilized: -1 image quality worsened, 0 image quality like before, 1 slight improvement, 2 improvement, 3 great improvement of image quality. Two different gradings were enquired, first improvement of the CNV visualization and second reduction of vessels or artifacts from other retinal layers (background noise/ window defect, false negative vessels from the choroid).

### Image analysis

**Figure 2:**
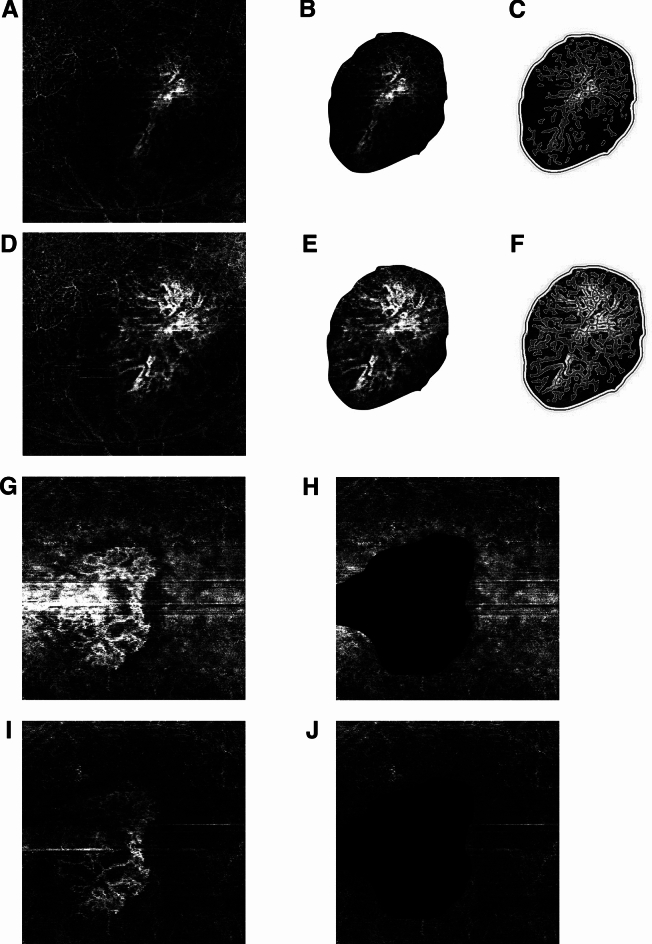
Preparation for image analysis. (**A**–**F**) One patient and (**G**–**J**) one patient. (**A**) Original image, (**B**) cropped image of CNV before BM correction and (**C**) with AngioTool vessel analysis. (**D**) Original image after 2 BM corrections (**E**) cropped image of CNV after 2 BM corrections, (**F**) cropped image of CNV after 2 BM corrections with AngioTool vessel analysis. (**G**) Uncorrected image, (**H**) uncorrected image with cropped out CNV with black filling, (**I**) image after 2 BM corrections, (**J**) image after 2 BM corrections with cropped out CNV with black filling.

The images were cropped with Photoshop (Adobe Inc., San Jose, California, USA), the exact same area of interest was delineated and cropped and the CNV area was cut out of the en face image. Leaving two parts for further analysis, 1. the CNV visible in the avascular slab and 2. the rest of the avascular slab minus the CNV, this area was filled with black. Figure 2A–F,G–J shows the steps of image preparation for ImageJ and Angio-tool analysis. Photoshop is licensed to UCSD and we used it crop images but we did not use it to enhance or alter any images.

**Figure 3:**
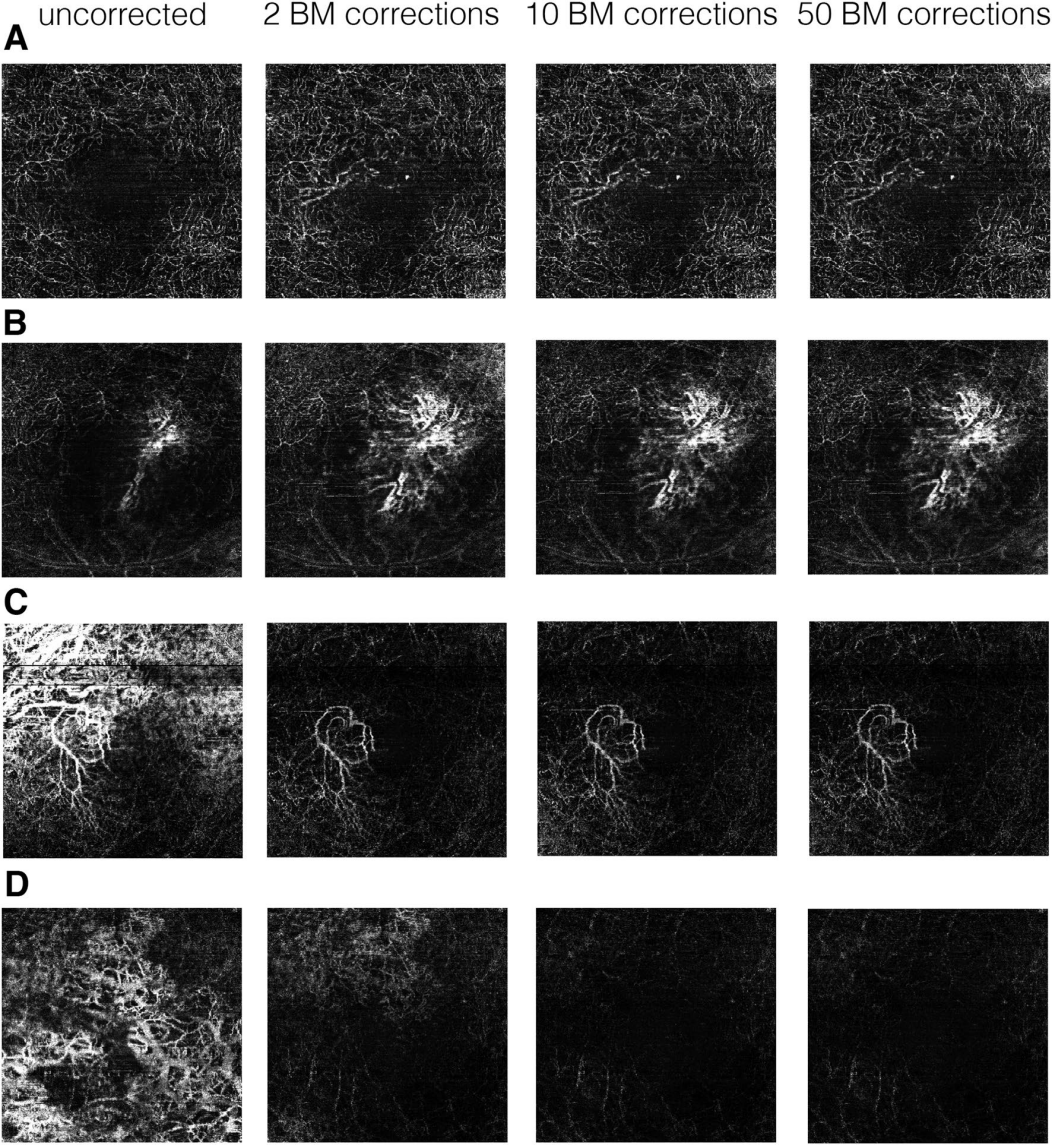
Examples of OCTA en face avascular slabs before and after manual corrections for BM. (**A**–**D**) represents each one patient, from left to right: uncorrected for BM segmentation errors, 2/512 manual BM corrections, 10/512 manual BM corrections, 50/512 manual BM corrections. (**A**) “New CNV”, after BM correction a CNV became visible. (**B**) “Improved CNV”, extend of CNV network improved. (**C**) “CNV visibility improved”, background noise reduced and thus CNV network visibility improved. (**D**) “Background noise reduced”, no CNV detectable after correction, large choroidal vessels visible on baseline image due to geographic atrophy disappeared after correction.

ImageJ (Image J v.1.53 k., Wayne Rasband, NIH, Bethesda, USA, http://imagej.nih.gov/ij) was utilized to measure the background changes. Higher numbers of arbitrary brightness units correlate with higher OCTA flow signal. The range is 0–255 of arbitrary units (AU). As we are measuring in the avascular layer, in the absence of pathology, the whole *en face* image should be black. Flow signal is only expected in areas of perfusion and a CNV should be visible in white. White in areas without CNV are considered background noise. The flow signal and bright areas of the vessels in the avascular layer can be visible in cases of false BM segmentation, when areas of atrophy are present, due to hypertransmission of the signal to choroid, the large choroidal vessels are often visible in the avascular layer, giving a false positive image of CNV (Fig. 3C). We analyzed the change of brightness intensity in both CNV and background of OCTA en face projection for each segmentation correction. Secondly, the ImageJ plug-in Angio-tool (v0.6a, National Cancer Institute, Center for Cancer Research, Bethesda, USA, https://ccrod.cancer.gov/confluence/display/ROB2/Downloads)^13^ was utilized to assess the CNV projection. The vessels' percentage area of the region of interest, was measured for every image before, after two, ten, and fifty BM corrections and then the change in percentage vessel density was compared between uncorrected and corrected images.

### Statistical analysis

Two human experts meticulously graded the segmentation-corrected images, subjecting them to correction cycles of 2, 10, or 50 times to pinpoint the most effective correction scheme. Each grader assigned a numerical value (− 1, 0, 1, 2, 3) to re-segmented images, evaluating the ease of identifying specific pathologies in comparison to the original images. These ordinal grades, representing the responses, were meticulously analyzed across various segmentation correction schemes and graders. The analysis employed a repeated measures ordinal regression model, implemented through the GENMOD procedure in SAS version 14.

In the case of data derived from ImageJ measurements of brightness intensity or Angio-tool measurements of vessel density in the region of interest, paired tests were meticulously conducted either using paired t-test or sign test depending on data distribution. These tests compared the original images with the segmentation-corrected images and were performed using JUMPER software version 17. P value of less than 0.05 (p < 0.05) was considered statistically significant.

## Results

Of the total 198 included OCTA’s, 125 did not need manual correction, either due to good CNV visualization or lack of incorrectly segmented BM in OCT. About 1/3 (73/198) of the total number of the scans showed major BM segmentation errors and were manually corrected for 2,10 or 50 BM of the 512 scans. After correction 55% (40) of the 73 corrected OCTA’s improved in image quality (CNV visualization and/or Background) improving the rate of good quality OCTA images from 63 to 83%.

### Expert grading

Inter-grader correlation: for expert grading on CNV easy identification, there was no statistically significant difference between the two expert graders, (p = 0.4165). Effect of number of corrected scans on OCTA image: two corrections showed the most significant improvement in CNV identification compared to baseline uncorrected image (2-correction median grade = 1 versus baseline median of zero, p < 0.0001. Multivariate repeated regression analysis demonstrated that there was no significant difference among segmentation correction schemes (2 vs. 10 vs. 50, p = 0.1386) though there was a trend for 10- or 50-correction being better than 2-corrections (10-correction median grade = 2 versus 2-correction median grade = 1, p = 0.055; or 50-correction median grade = 2 versus 2-correction median grade = 1, p = 0.081). There was no significant difference between 10- and 50-correction at all (p = 0.781).

Figure 3A shows an example of a CNV that became visible after 2 BM corrections, that was not visible on the *en face* image before. In the case below (Fig. 3B) the extent of the CNV network improved. In both examples subretinal fluid (SRF) was a main factor for wrong BM segmentation, here the BM was falsely shifted anterior of the SRF.

For human expert grading on image background, again there was no statistically significant difference between the two expert graders, (p = 0.203). However, background grades were significantly different among the three segment-correction schemes (p = 0.0005). Background grading on images with 2-correction (median grade = 1.5) was significantly lower than that of images with either 10- (median grade = 2, p = 0.0078) or 50-correction (median grade = 2, p < 0.0001). No difference was noted between 10- and 50-correction schemes (p = 0.330).

### Image J analysis for background intensity measurement

**Figure 4:**
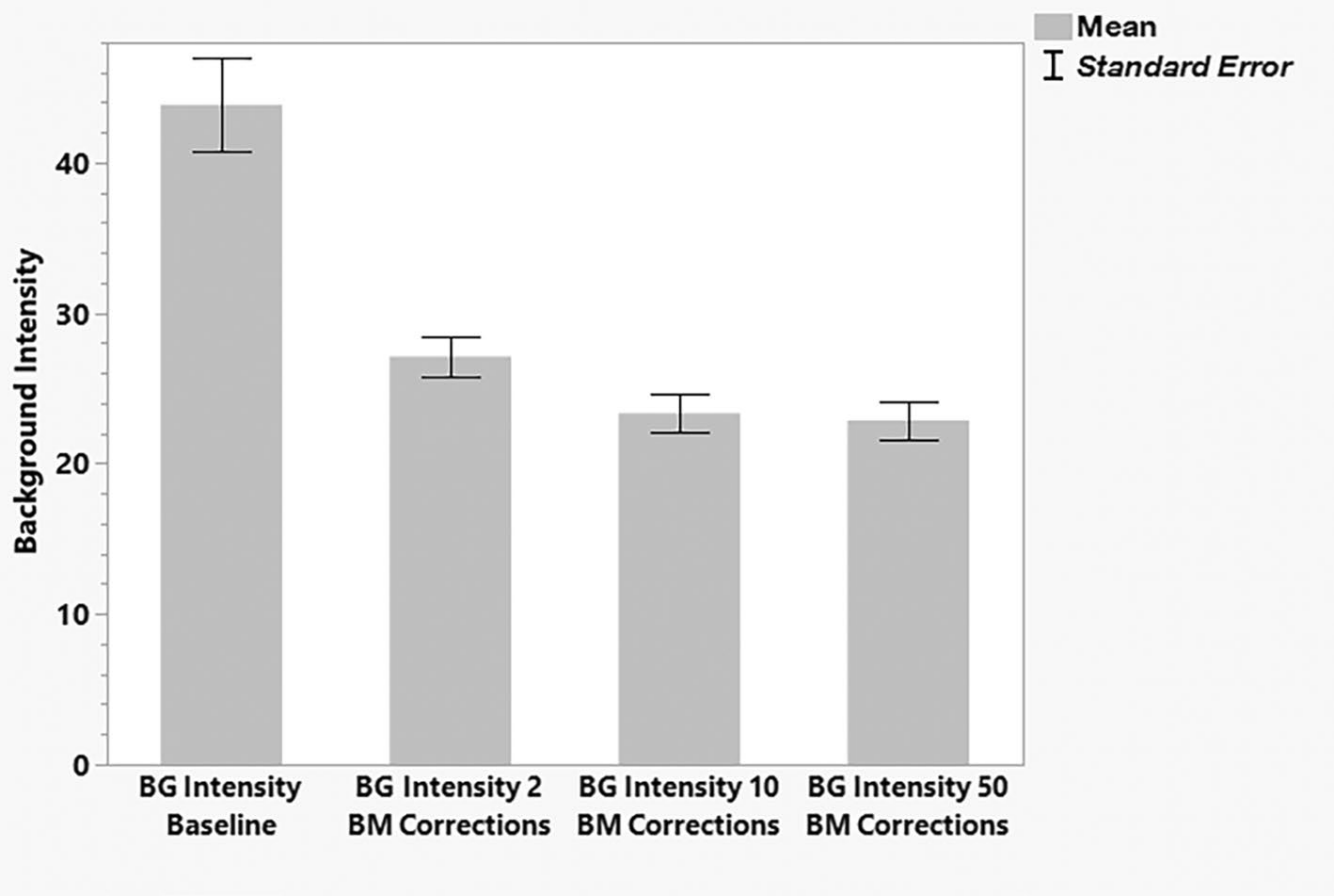
The bar graph shows mean background intensity measurement using Image J for baseline, background intensity after 2 corrections, after 10 and after 50 corrections.

We used matched pair tests, the minimum correction number of 2 shows a significantly reduced background noise from the baseline (Fig. 4).

The mean baseline intensity was 43.86 arbitrary units (AU) and was reduced to 27.12 after 2 corrections (16 AU reduction, p < 0.001, sign test. Both correction of ten and correction of 50 B scans further reduced background intensity by 3.76 AU (p < 0.0001) and by 4.25 AU (p < 0.0001) from 2-correction; however, the reduction magnitude was limited.

**Figure 5:**
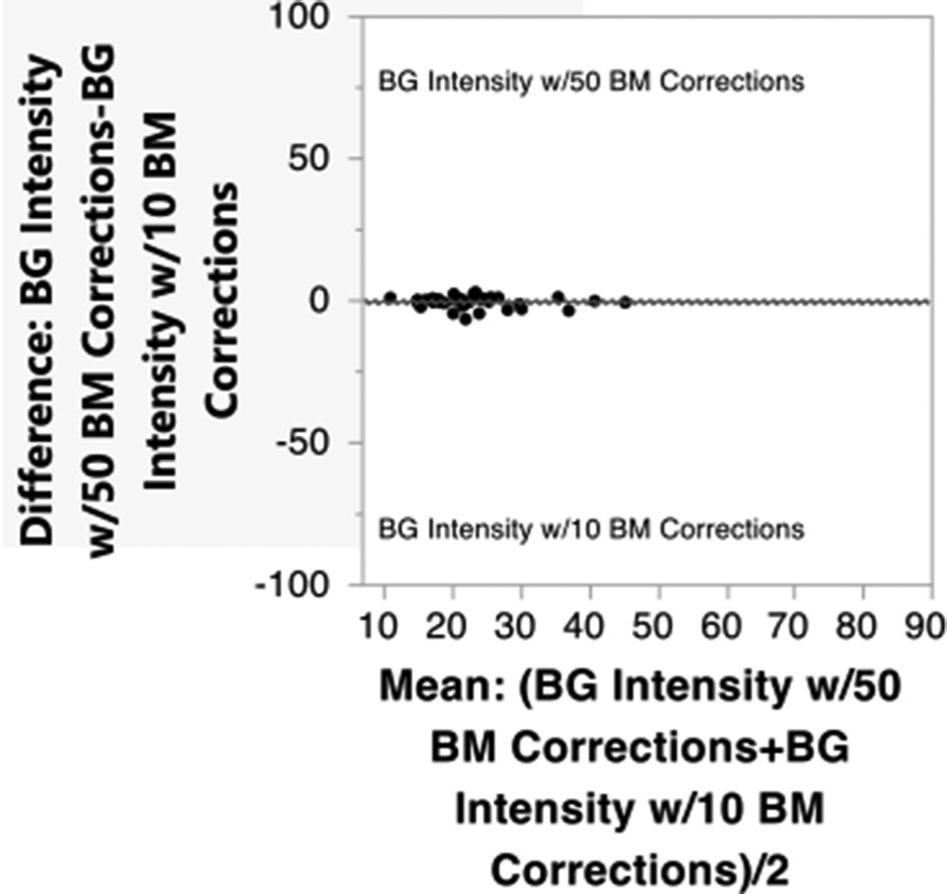
Mean background intensity after 50 corrections compared to after 10 corrections measured in Image J.

50 corrections did not significantly improve the background quality compared to 10 corrections (p = 0.152, mean reduction from 23.36 to 22.867, not significant (Fig. 5).

### Angio-tool analysis results for vessel density

The data was normally distributed, therefore we used a matched test. We looked at the change of vessel density after each correction scheme compared to baseline and we also compared the different correction schemes between each other.

We observed statistically significant changes in vessel density after 2, 10, and 50 corrections compared to baseline, uncorrected images (p values 0.0412, 0.0106, 0.0095 respectively). The analysis between corrections showed a statistically significant change in vessel density after 10 corrections compared to 2 corrections (p = 0.0432, mean change from 31.2078 to 29.5457). There was a borderline trend towards statistical significance, with a small change in vessel density measurements between 50 and 2 corrections (p = 0.0616, mean change from 31.2078 to 29.2997).

Overall, we did not observe an increase in AngioTool CNV vessel density percentage area measurements after manual correction. A subgroup of images shows an increased area of vessels in the analysis but for the second half, this improvement got lost in the reduction of flow signal around the actual CNV. An example, (Fig. 3C) the uncorrected image shows overlap of background noise and CNV, making a clear distinction of CNV difficult. After two, ten or fifty corrections of BM, this overlap is eliminated and the CNV is easier to appreciate for the human eye. In this example, vessel density calculated by Angio-tool is decreased after correction, because less signal (white pixels) is measured. Table 1 shows change in mean background intensity measured in Image J and vessel density measured in Angio-tool depending on number of corrections.

**Table 1:** Change in mean background intensity measured in Image J and vessel density measured in Angio-tool depending on number of corrections of Bruch’s Membrane (BM) segmentation errors.

Number of corrections	0 (baseline)	2	10	50
Image-J (AU) mean ± SD	43.86 ± 18.37	27.12 ± 7.88	23.36 ± 7.62	22.87 ± 7.40
Angio-tool (%) mean ± SD	33.93 ± 11.01	31.21 ± 10.97	29.55 ± 11.57	29.30 ± 12.16

## Discussion

OCTA imaging technology is still developing and evolving, and technical limitations and artifacts associated with OCTA can also affect the accuracy of image interpretation, particularly in patients with advanced AMD. Larger studies could address the factors causing segmentation errors. Other authors studied the prevalence of segmentation errors in retinal diseases in OCTA and found that the highest percentages of inaccurate segmentation were observed in the nAMD group (90.1%). This is due to the presence of pigment epithelium detachment (PED), intraretinal (IRF) and subretinal fluid (SRF) associated with this disease^10^. It is our impression that the more distorted the anatomy, the higher the chance of segmentation error. Also scans with larger CNV lesions may need more manual correction of segmentation than smaller lesions, which is consistent with our observations. Overall, while OCTA has shown great promise in the diagnosis and monitoring of AMD, accurate interpretation of OCTA images in the context of this complex disease requires specialized training, expertise, and careful attention to potential sources of error.

Our results of human expert grading and examples in Fig. 3. demonstrate that even minimal correction of 2 BM lines significantly improves the quality of OCTA CNV projection and removes the background noise in eyes with CNV and segmentation errors. Our analysis showed that there is further improvement after 10 B scan line corrections of Bruch’s Membrane manually compared to 2 corrections, but the magnitude of this improvement is small and likely not to be of clinical importance. Doing 50 manual corrections improves background noise slightly but not vessel visualization and is not recommended.

Minimal (2 corrections) is already sufficient to markedly improve image quality (CNV and background/false positive). In particular, we have observed a significant improvement in decrease of background intensity measured by Image J already after 2 corrections of BM (Fig. 4).

This may help to make quick improvements in the decision-making in a clinical setting and improve the longitudinal follow-up of patients, achievable for both ophthalmologists and technical staff. Within one minute, substantial improvement can be achieved.

We used and recommend BM correction as reported by others because it can be corrected due to the ability to visualize it even in cases of severe pathology^12^ and even two B-scan resegmentations (minimal manual correction) helped the propagation software to fix these issues. In areas of atrophy, the segmentation is often shifted posteriorly and thus the images include the choroidal vasculature. Even larger areas of atrophy were improved with our minimal correction approach making the false positive large choroidal vessels disappear from the final corrected avascular en face projection (Fig. 3D). In eyes with multiple small areas with neighboring falsely segmented retinal layers, with correctly segmented areas in between, more corrections were helpful. This was the case where multiple poorly segmented areas of geographic atrophy or drusen.

The improvement of CNV visualization as well as the reduction of background signal was quantified by the human expert. In our work, we observed a discrepancy between the expert grader and the automated AngioTool measurements. We found that AngioTool is suboptimal when used alone as it has difficulties differentiating between artifacts (background noise) and real CNV vessels. Coscas et al.^14^ in agreement with our study, concluded that vessel density is an inferior biomarker for the valid detection of exudative CNV lesions. Therefore, for measurements with Angio-tool, we looked at the change in vessel density and not at the sign of change (increased + or decreased −), because the interpretation of the image requires human expertise. In cases where vessel density decreased, measured in Angio-Tool we observed less signal (white pixels of the image) because of the elimination of false positive vessels from choroid due to atrophy and background vessels from projection artifacts. In this case, the decrease in vascular percentage is expected and makes sense to human observers. In cases of clearly improved CNV image, we would expect increased vascular percentage area, but overall it may be still decreased because even though the CNV is more visible or easier to distinguish the background vessels and false positive “vessels” will disappear making a total measured signal by Angio-Tool decreased compared to the uncorrected baseline image.

We have demonstrated the importance of checking the automated segmentation for possible errors and we have proven that in some cases with minimal manual correction of these errors, we can achieve better overall quality of the image and improve CNV visualization. This is a task that can be swiftly executed and holds substantial potential to enhance image interpretation for retina physicians. In order for retina specialists to effectively leverage OCTA for visualizing vascular patterns and treatment-related changes, it is imperative to correct image segmentation; otherwise, the imaging results can become highly variable.

Segmentation-corrected OCTA may offer better detection of choroidal neovascularization and follow-up of changes in CNV morphology in response to anti-VEGF treatment. The need for improvement of automated software segmentation in eyes with pathology has been recognized and there are methods using neural networks studied by our group to refine the segmentation in OCT and OCTA^15,16^. In our study, we only corrected Bruch’s Membrane, as suggested by other authors^12^, although errors in other layers may be present and might not be relevant for this disease, future work could involve full segmentation correction of all layers, but this would be very laborious to do manually and might require AI methods to help with automate this task. We note that in our study the segmentation correction was not compared between type 1 and type 2 CNV or in different stages (active, resolving, healed) of CNV. Rather we have shown that reduced background noise and improved CNV identification in OCTA can be achieved with the minimal manual segmentation correction, with the most substantial improvement after two corrections compared to baseline uncorrected images.

It is important to emphasize, that OCTA is not critical in the management of CNV and OCT is still the gold standard for decision making. OCT B-scan and information about the fluid combined with history and visual acuity usually suffice. However, OCTA is the only modality that allows us to visualize the CNV network and even non-invasively monitor its changes over time^17^. OCTA may be useful in the management of complex cases, distinguishing atypical cases of CNV and in other situations. When using OCTA, manual segmentation correction is crucial for image interpretation to reduce the chance of false positive and false negative results. Propagation software in the Heidelberg device permits better quality images of the CNV vessels even after correction of Bruch’s membrane in only 2 B-scans.

Limitations of our study and methods include the fact that the propagation software we studied is only available for the Heidelberg Spectralis. Small segmentation errors limited to a few scans might not be corrected by propagation software using only 2 corrected lines and correction of a large number might improve such errors but is time consuming.

## Data Availability

The datasets generated and/or analyzed during the current study are not publicly available due to the possibility of patients’ identification from retinal imaging of OCTA vascular patterns but are available from the corresponding author on reasonable request.
